# Development and internal validation of a multimodal deep learning model for screening-level lower-limb alignment using plantar 3D foot scans

**DOI:** 10.1371/journal.pdig.0001802

**Published:** 2026-11-11

**Authors:** Simin Ni, Yaxi Chen, Chenyan Wang, Ding Zhou, Jiaqi Liu, Mo Zhou, Julian Leong, Yipeng Hu, Jie Huang, Jinwu Wang, Chaozong Liu

**Affiliations:** 1 Division of Surgery and Interventional Science, University College London, London, United Kingdom; 2 Mechanical Engineering Department, University College London, London, United Kingdom; 3 UCL Hawkes Institute, University College London, London, United Kingdom; 4 College of Integrated Circuits and Micro-Nano Electronics, Fudan University, Shanghai, China; 5 Department of Orthopedic Surgery, Shanghai Ninth People’s Hospital, Shanghai Jiao Tong University School of Medicine, Shanghai, China; 6 Department of Medical Physics and Biomedical Engineering, University College London, London, United Kingdom; University College London, UNITED KINGDOM OF GREAT BRITAIN AND NORTHERN IRELAND

## Abstract

Lower-limb alignment is a key determinant of musculoskeletal loading and is associated with biomechanical risk pathways for conditions such as knee osteoarthritis and plantar fasciitis. However, routine alignment assessment still relies on radiography or specialist clinical examination, limiting scalability for early screening. Here, we investigated whether plantar foot morphology captured by weight-bearing 3D scanning can serve as a computational screening feature for proximal lower-limb alignment, and we developed a multimodal deep learning framework to support scalable alignment screening. We established a cross-sectional dataset of 167 participants (adults and children aged 6–60 years) with standardised plantar 3D foot scans and clinical labels for arch type, hindfoot alignment, and frontal-plane knee alignment. The proposed model jointly learns from plantar scan images and 16 geometric morphological features, integrated using feature-wise attention to prioritise alignment-relevant cues across modalities. Performance was evaluated using accuracy, precision, recall, F1 score, and AUC on a held-out test set, and interpretability was assessed with class activation mapping (CAM). Ablation studies compared alternative image encoders and fusion variants, including image-only, morphology-only, and fusion without attention. The attention-based multimodal model showed competitive but task-dependent performance, achieving accuracies of 89% for arch type, 81% for hindfoot alignment, and 76% for knee alignment. The image-only ViT achieved the highest accuracy for arch classification, while the ViT without-attention configuration achieved slightly higher accuracy and macro-F1 for knee alignment. These findings indicate that the benefit of multimodal attention fusion varied across alignment tasks. Activation maps consistently highlighted clinically relevant plantar regions associated with alignment patterns, suggesting morphological correlations consistent with a distal-proximal kinetic chain hypothesis. Together, these findings demonstrate the potential of plantar 3D scanning combined with multimodal, explainable deep learning as a non-invasive approach for screening alignment-related biomechanical risk factors, warranting further external validation before clinical translation.

## Author summary

Lower-limb alignment influences how forces are distributed during standing and walking, and abnormal alignment is associated with common conditions such as knee osteoarthritis and foot pain, yet routine assessment often relies on radiographs or specialist examination, which limits scalable early screening. To address this, we collected weight-bearing plantar 3D foot scans from 167 participants with clinical labels for arch type, hindfoot alignment, and frontal-plane knee alignment, and developed a computer model that combines information from plantar scan images and quantitative foot-shape measurements to classify these alignment outcomes. We found that integrating both sources of information showed competitive performance across all three tasks, and the model consistently highlighted clinically relevant plantar regions linked to alignment patterns. These findings indicate that weight-bearing plantar 3D scanning can serve as a non-invasive digital marker of proximal lower-limb alignment and may support scalable screening to enable earlier orthotic or preventive interventions for alignment-related biomechanical risk.

## Introduction

Lower-limb alignment denotes the orientation of the hip, knee, ankle, and foot relative to the mechanical axis in the coronal, sagittal, and axial planes [1]. Physiological alignment maintains postural stability, economical gait, and balanced joint loading. At the knee, frontal-plane malalignment (varus or valgus) alters tibiofemoral load distribution and accelerates cartilage degeneration; varus configuration increases osteoarthritis risk, and both varus and valgus are associated with faster structural progression [2,3]. Patients with knee osteoarthritis (KOA) exhibit greater alignment variability and more pronounced deformity than asymptomatic controls [4].

Alignment should be interpreted across the kinetic chain (the interdependent system of joints and segments through which forces are transmitted from foot to hip). Hindfoot position commonly compensates for knee mechanics: displacement of the knee mechanical axis is frequently offset by opposite calcaneal alignment [5], and women with varus knees often demonstrate hindfoot valgus [6]. These interactions influence clinical assessment and surgical planning, yet the directionality and the role of hindfoot mobility remain uncertain. Distal structure further contributes: pes planus, characterised by medial longitudinal arch collapse with hindfoot valgus and forefoot abduction, is prevalent in adults [7] and associates with altered gait and load transfer involving the foot, knee, and lumbar spine [8,9]. Midfoot morphology, including navicular height, correlates with symptom severity in medial compartment disease [10], and further evidence suggests an association between KOA and distal foot structural features [11]. These observations collectively motivate examining arch type, hindfoot alignment, and frontal-plane knee alignment as an integrated set of clinically relevant targets, reflecting the hierarchical organisation of the lower-limb kinetic chain from distal to proximal structures.

Despite this relevance, routine assessment relies on subjective examination or radiography, which requires equipment and exposes patients to ionising radiation [12]. Automated radiograph-based systems improve measurement consistency but do not eliminate these constraints [13–17]. In parallel, weight-bearing 3D foot scanning provides reproducible geometric features to support orthotic design [18], insoles can modify load distribution and alleviate symptoms [19], and machine learning models based on imaging-derived features have shown promise for applications in lower-limb musculoskeletal conditions [20–22]. The integration of imaging-based and morphological data for clinical classification tasks has been increasingly explored across musculoskeletal and cardiovascular domains [23–25]. However, existing studies rarely attempt to infer multi-level lower-limb alignment across the kinetic chain from foot data alone. Because alignment reflects interdependent interactions across distal and proximal structures, integrating complementary image-based and morphological inputs within an interpretable framework is a natural design requirement that prior single-modality work has not addressed. While plantar scanning is established for orthotic design, its potential as a non-invasive surrogate for proximal alignment assessment remains largely unexplored.

In this single-centre prospective observational study, we investigated whether plantar 3D foot morphology can serve as a computational screening feature for proximal lower-limb alignment and proposed a multimodal deep learning framework to perform multi-class classification of arch type, hindfoot alignment, and frontal-plane knee alignment using weight-bearing plantar scans and derived structural features, with internal validation on a held-out test set. We developed an attention-based multimodal deep learning framework that integrates plantar image embeddings with geometric morphology features to adaptively emphasise alignment-relevant cues across modalities. Model design choices are examined via benchmarking across multiple image backbones (Inception, ResNet, EfficientNet, and Vision Transformer) and comparisons with morphology-based baselines (XGBoost, Random Forest, multilayer perceptron, and Logistic Regression), alongside ablation variants including image-only, morphology-only, and multimodal fusion without attention. We further apply CAM to localise plantar regions driving model decisions, supporting transparent alignment screening and potential translation to personalised orthotic and insole design. The main contributions are:

- We established a standardised weight-bearing plantar 3D foot scanning dataset with clinically assessed labels for arch type, hindfoot alignment, and coronal knee alignment.
- We proposed a novel multimodal deep learning model with feature-wise attention fusion for automated screening of multi-level lower-limb alignment.
- Our proposed attention-based multimodal model achieves competitive overall performance across tasks, outperforming end-to-end deep learning approaches and conventional morphology-based baselines.
- We provided explainable predictions using CAM to highlight clinically relevant plantar regions associated with different alignment patterns, supporting the development of personalised orthotics for malalignment correction.

## Materials and methods

### Ethics statement

The study involving human participants was reviewed and approved by the Institutional Review Board of Shanghai Jiaotong University School of Medicine (approval no: SH9H-2022-T125-2) and by the Ethics Committee of HRA and Health and Care Research Wales (HCRW) (REC Ref No. 25/SC/0250). Written informed consent to participate in this study was provided by the participants.

### Data collection

As a single-centre prospective observational study, we consecutively enrolled outpatients from October 2023 to September 2024 at the 3D Printing Centre of the Ninth People’s Hospital (Shanghai, China), a clinical facility providing patient-specific 3D printing services, including surgical planning and personalised orthotic fabrication, where weight-bearing foot scanning is routinely performed as part of orthotic assessment. All participants provided written informed consent before any procedures. Fig 1 summarises the recruitment pathway and scanning workflow.

**Fig 1:**
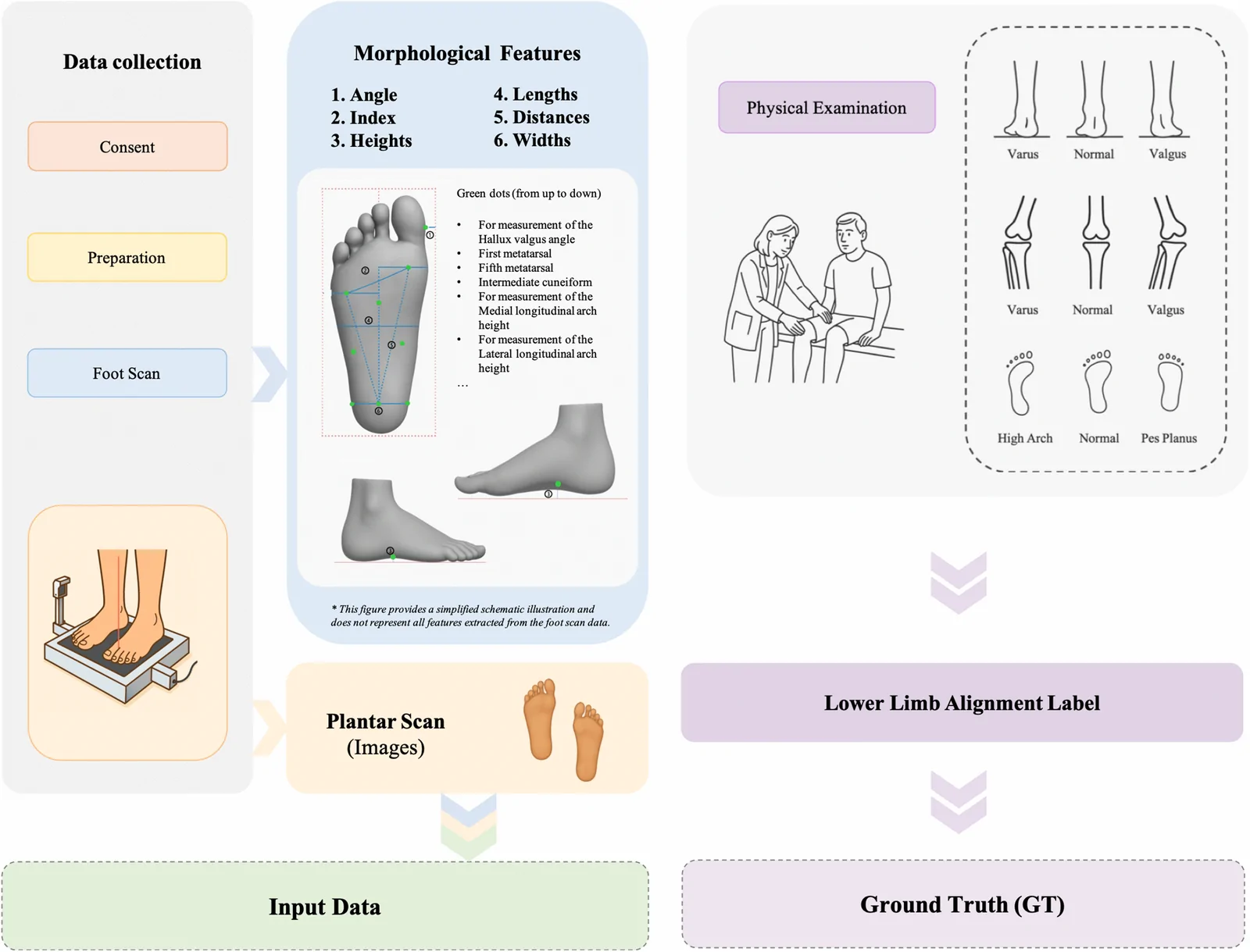
The data collection workflow involved acquiring static foot scans, extracting features from 3D foot scans, annotating alignment categories by clinicians, and assembling the dataset.

Eligibility was age 6–60 years, with or without clinically observed lower-limb malalignment. This range was selected to reflect the clinical population attending the 3D Printing Centre, encompassing both paediatric and adult patients in whom alignment screening is clinically relevant; a recent referral sample from the centre had a mean age of 33.9 years (SD 17.2). Exclusion criteria were (i) lower-limb surgery within the previous 12 months; (ii) acute musculoskeletal injury at enrolment; and (iii) neurological or vestibular disorders that could affect gait or posture. Participants were required to stand independently during scanning.

Each participant underwent static, full weight-bearing 3D foot scanning (Model 1.0, BlackFlame Science and Tech., Shanghai), yielding plantar images and 16 morphological features; sex was recorded as a demographic covariate (variables listed in Table 1). These 16 measurements were selected to capture key geometric dimensions of plantar foot structure—including arch height, foot length, width, and angular deformity—that have been associated with lower-limb alignment and biomechanical loading patterns [20–22]. Arch type was classified into Pes planus, neutral, and high arch categories using standardised clinical assessment. Frontal-plane knee and hindfoot alignment were classified as varus, neutral, or valgus based on standardised clinical examination of knee alignment and calcaneal position relative to the tibial axis during weight-bearing stance, consistent with established protocols and published reference values [26–28]. All classifications were performed by two independent examiners. Inter-rater agreement between the two independent examiners was almost perfect for hindfoot, knee, and arch classification (Cohen’s κ=0.873, 0.910, and 0.907, respectively) [29]; discrepant cases were resolved by a third examiner. We acknowledge that clinical examination does not provide the angular precision of full-leg standing radiography, which remains the gold standard for coronal-plane alignment measurement; however, clinical examination is widely used in screening contexts and was considered appropriate for the purposes of this study. To minimise collinearity and potential label leakage, the scan-derived Arch Index, which is highly correlated with clinical arch class, was excluded from model inputs for the arch task. No equivalent exclusion was required for the hindfoot or knee alignment tasks, as none of the remaining 16 morphological features demonstrated a comparably direct definitional relationship with the hindfoot or knee alignment labels.

**Table 1:** Foot morphology features grouped by measurement type.

Type	Measurement	Unit	Brief definition
**Angle**	Hallux valgus angle	deg	Angle between the 1st metatarsal axis and proximal phalanx of the hallux.
**Index**	Arch Index	–	Ratio of midfoot contact area to total footprint (excluding toes).
**Heights**	Medial arch height	mm	Vertical height of the medial arch above the support plane.
**Heights**	Regional arch heights	mm	Heights at predefined foot regions.
**Heights**	Lateral malleolar height	mm	Vertical height of the lateral malleolus above the support plane.
**Lengths**	Total foot length	mm	Posterior heel point to most distal toe.
**Lengths**	Arch length	mm	Heel to arch apex/navicular along foot axis.
**Lengths**	Medial midfoot length	mm	Heel to medial midfoot landmark along the medial column.
**Lengths**	Lateral midfoot length	mm	Heel to lateral midfoot landmark along the lateral column.
**Lengths**	Lateral column (dorsolateral) length	mm	Calcaneus to 5th metatarsal head along the lateral column.
**Distances**	Medial arch–heel distance	mm	Heel centre to medial arch apex (planar).
**Distances**	Lateral arch–heel distance	mm	Heel centre to lateral arch apex (planar).
**Widths**	Forefoot width	mm	Maximum width across metatarsal heads.
**Widths**	Heel width	mm	Maximum transverse width of the calcaneal region.
**Widths**	Midfoot (tarsal) width	mm	Maximum transverse width across the midfoot region.
**Widths**	First–fifth metatarsal head width	mm	Distance between the 1st and 5th metatarsal heads.

The scan-derived Arch Index was excluded from model inputs for arch task to reduce collinearity and avoid label leakage due to its strong correlation with clinical arch classification.

### Model architecture

We proposed a multimodal deep learning framework that integrates plantar scan images with structured clinical measurement data for alignment classification, as illustrated in Fig 2. The architecture comprises three key components: (*i*) an image feature encoder, (*ii*) a morphological feature encoder, and (*iii*) an attention-based fusion module that adaptively combines the two modalities for downstream classification.

**Fig 2:**
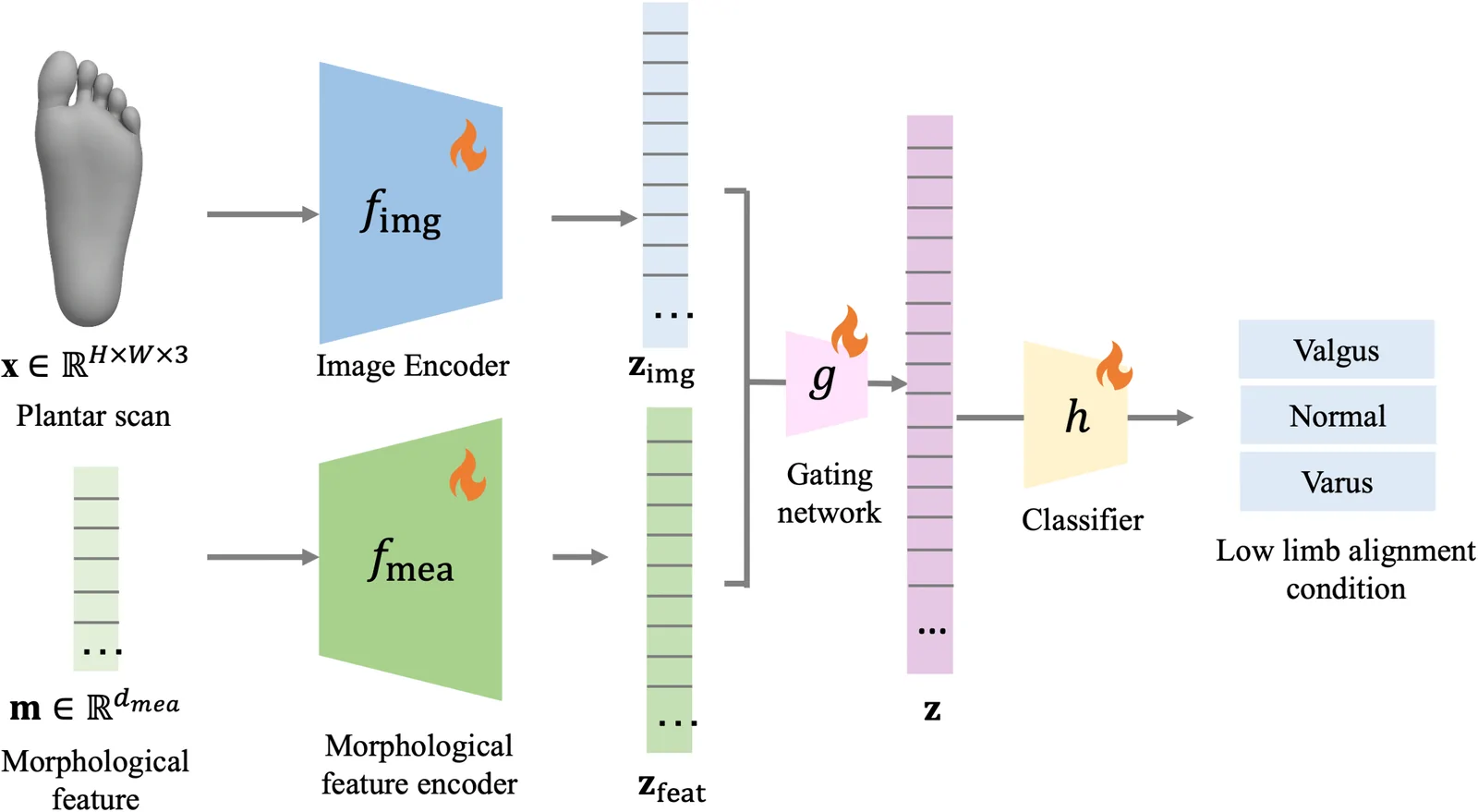
Overview of the proposed framework, illustrated using the knee alignment classification task as an example. The same architecture is applied to the other two tasks.

#### Image feature and morphological feature extraction.

Let $𝒳\subset {\mathbb{R}}^{H\times W\times 3}$ denote the RGB image space, and let $ℳ\subset {\mathbb{R}}^{d_{mea}}$ denote the space of morphological features extracted from clinical measurements, where *d*_mea_ is the number of morphological features.

Given a dataset $𝒟=\{(x_{i},m_{i},y_{i}){\}}_{i=1}^{N},$ each sample consists of an image $x_{i}\in 𝒳$, its corresponding morphological feature vector $m_{i}\in ℳ$, and a classification label $y_{i}\in 𝒴=\{1,2,3\}$, representing the three alignment categories. In the following sections, we denote the image and morphological feature inputs as $𝐱=x_{i},$ $𝐦=m_{i}.$

An image encoder $f_{img}(\cdot ;\;\theta_{img}):𝒳\to {\mathbb{R}}^{d_{img}},$ parameterised by $\theta_{img}$, maps an input image $x_{i}$ to a *d*_img_-dimensional embedding:


$$\begin{array}{c} {𝐳}_{img}=f_{img}(𝐱;\theta_{img})\in {\mathbb{R}}^{d_{img}}. \end{array}$$ (1)

Similarly, a feature encoder $f_{mea}(\cdot ;\;\theta_{mea}):ℳ\to {\mathbb{R}}^{d_{feat}},$ parameterised by $\theta_{mea}$, projects the morphological feature vector **m** from ${\mathbb{R}}^{d_{mea}}$ into a *d*_feat_-dimensional latent space:


$$\begin{array}{c} {𝐳}_{feat}=f_{mea}(𝐦;\theta_{mea})\in {\mathbb{R}}^{d_{feat}}. \end{array}$$ (2)

#### Feature-Wise attention fusion.

The image and feature embeddings are first concatenated to form a joint representation:


$$\begin{array}{c} {𝐳}_{cat}=[\;{𝐳}_{img},\;{𝐳}_{feat}\;]\in {\mathbb{R}}^{d_{img}+d_{feat}}. \end{array}$$ (3)

To adaptively reweight the concatenated features, a feature-wise attention (gating) mechanism is applied. Specifically, a gating network $g(\cdot ;\;\psi_{g}):{\mathbb{R}}^{d_{img}+d_{feat}}\to (0,1{)}^{d_{img}+d_{feat}},$ parameterised by $\psi_{g}$, produces a weight vector:


$$\begin{array}{c} 𝐰=g({𝐳}_{cat};\psi_{g})\in (0,1{)}^{d_{img}+d_{feat}}. \end{array}$$ (4)

The final fused representation is then obtained through element-wise modulation of the concatenated vector:


$$\begin{array}{c} {𝐳}_{fuse}=𝐰⊙{𝐳}_{cat}\in {\mathbb{R}}^{d_{img}+d_{feat}}. \end{array}$$ (5)

#### Downstream classification.

The fused representation is passed through a classifier $h(\cdot ;\;\gamma_{cls}):{\mathbb{R}}^{d_{img}+d_{feat}}\to {\mathbb{R}}^{3},$ parameterised by $\gamma_{cls}$, which maps the fused embedding to the output logits:


$$\begin{array}{c} \hat{𝐲}=h({𝐳}_{fuse};\gamma_{cls})\in {\mathbb{R}}^{3}. \end{array}$$ (6)

The classifier $h(\cdot ;\;\gamma_{cls})$ is implemented as a two-layer Multilayer Perceptron (MLP) with ReLU activation.

#### Optimization.

All model parameters $\left\{\theta_{img},\;\theta_{mea},\;\psi_{g},\;\gamma_{cls}\right\}$ are jointly optimised. The model is trained to minimise the weighted cross-entropy loss:


$$\begin{array}{c} ℒ=-\sum_{c=1}^{3}\omega_{c}\;y_{c}\log{\hat{y}}_{c}, \end{array}$$ (7)

where $y_{c}\in \{0,1\}$ denotes the one-hot encoded ground-truth label for class *c*, and ${\hat{y}}_{c}$ represents the predicted probability of class *c* obtained via a softmax function applied to the output logits. The class-specific weights $\omega_{c}$ are computed using inverse frequency weighting from the training data to mitigate class imbalance. Equation (7) is optimised via gradient descent using the AdamW (Adam with decoupled weight decay regularisation) optimiser, jointly updating all network parameters.

### Experimental setup

The image encoder *f*_img_ is instantiated using a pretrained visual feature extractor (convolutional neural network or vision transformer architecture) with the task-specific classification head removed, ensuring that **z**_img_ captures high-level semantic representations without task-specific bias. We evaluate our framework using four pretrained visual backbones: Inception, ResNet, EfficientNet, and Vision Transformer (ViT). These four backbones were selected to represent a diverse range of architectural paradigms, enabling a comprehensive and fair comparison across model families. To ensure a fair comparison, all backbones were initialised with ImageNet pretrained weights, trained under identical optimisation settings, and evaluated on the same held-out test set using the same evaluation metrics.

The morphological feature encoder *f*_mea_ is implemented as a MLP composed of fully connected layers with non-linear activations. The fusion gate *g* consists of a learned linear mapping followed by a sigmoid activation to produce feature-wise attention weights. The classifier *h* consists of a two-layer MLP with ReLU activation.

The dataset was divided into 60% training, 20% validation, and 20% test sets using a fixed split stratified by class label to preserve label distribution across sets. To estimate uncertainty due to finite test-set sampling, non-parametric bootstrap resampling was performed on the held-out test predictions with 100 resamples. For each resample, performance metrics were recomputed. Results are reported as mean ± standard deviation across bootstrap resamples, while the corresponding 95% confidence intervals are provided in the Supporting Information. Analysis was performed at the foot/scan level, as each plantar scan represented a limb-specific morphological observation with its own corresponding clinical alignment label. The dataset was therefore split and analysed at the scan level, with stratification by class label to preserve label distribution across sets. We acknowledge that bilateral scans from the same participant are not fully independent; however, left and right feet may differ in alignment labels and morphological measurements, supporting their treatment as separate screening targets in this study. As a sensitivity analysis, we also performed a strict participant-level split in which all scans from the same participant were assigned to the same training, validation, or test partition. This analysis used the structured morphology features to assess whether predictive performance was retained after removing participant overlap across data partitions.

Image preprocessing includes resizing, normalisation, and stochastic data augmentation ($\pm 10^{\circ }$random rotation and horizontal flipping) applied only to the training set. Validation and test images are resized and normalised without augmentation. The image encoder is initialised with ImageNet-pretrained weights, and the input resolution is adjusted according to the selected backbone architecture.

Models were trained using mini-batches of size 32. The AdamW optimiser was used with a weight decay of $1\times 10^{-3}$ and an initial learning rate of $5\times 10^{-5}$. The learning rate was reduced by a factor of 0.5 when the validation loss did not improve for three consecutive epochs. Additional optimisation hyperparameters and candidate values are provided in S1 Table.

Class weights $\omega_{c}$ were incorporated into the cross-entropy loss to mitigate class imbalance. Early stopping and model checkpoint selection were applied based on the validation macro-averaged F1 score (i.e., the unweighted mean of per-class F1 scores), rather than accuracy, to reduce the influence of majority-class dominance. Training was terminated if no improvement is observed for 50 consecutive epochs. All reported results were obtained from the held-out test set using the checkpoint that achieves the highest validation macro-F1 during training.

### Comparison and ablation study

To comprehensively evaluate the proposed multimodal attention fusion framework, we compared its performance against three baseline configurations: (1) morphological features only, (2) image only, and (3) image–morphological feature fusion without attention. These baselines isolate the contributions of each modality and the effect of the attention-based fusion mechanism.

### Morphological features only baseline

As a baseline, we trained classical machine learning models (including Random Forest, XGBoost, Logistic Regression, and MLP) using only numerical foot morphology features. These models independently predicted categorical outcomes for arch type, hindfoot alignment, and knee alignment, serving to isolate the predictive potential of morphological data alone.

### Image only baseline

In this baseline, only plantar scan images were provided as input to isolate the predictive capacity of visual features. The image branch was trained in an end-to-end manner using four pretrained backbones: Inception, ResNet, EfficientNet, and ViT. No morphological or demographic data were included.

### Image and morphological features without attention fusion

To assess the benefit of multimodal integration, we implemented a dual-input baseline that jointly processed plantar scan images and morphological feature vectors. The image branch used the same pretrained encoders described in the Experimental Setup section, with the final classification head removed to produce image embeddings. These embeddings were concatenated with a 16-dimensional morphological feature vector and passed through a fully connected network, followed by a linear classification layer. This configuration represents a late-fusion approach, as no attention mechanisms or explicit feature-interaction modules were applied.

### Evaluation metrics

Model performance was evaluated using standard classification metrics, including accuracy, precision, recall, F1-score, and area under the ROC curve (AUC), to assess predictive accuracy across alignment categories. All multi-class metrics (precision, recall, F1, and AUC) are reported as macro-averaged values (i.e., the unweighted mean across all three classes), which treats each class equally regardless of its frequency and provides a more informative evaluation under class imbalance. These metrics were computed on the held-out test set, with final evaluation based on the model checkpoint that achieved the highest validation performance during training. AUC provided a threshold-independent measure of class separability, while F1-score offered a balanced view of precision and recall, particularly for imbalanced classes. Model performance was compared descriptively using held-out test metrics and bootstrap confidence intervals.

### Statistical analysis

Categorical variables (arch type, hindfoot alignment, knee alignment) were cross-tabulated and tested for association using Pearson’s $\chi^{2}$ test (α=0.05). To preserve participant-level independence, one foot was randomly selected from each participant with identifiable bilateral scans, yielding an inferential sample of *N* = 165 participants. For each contingency table, the degrees of freedom were calculated as (number of row categories −1) × (number of column categories −1). Effect sizes were summarised using Cramér’s *V*. Expected cell counts were inspected to assess the adequacy of the $\chi^{2}$ approximation; when any expected cell count was < 5, a Monte Carlo permutation test for independence (100,000 permutations, two-sided) was performed as a sensitivity analysis and its *p*-value reported. To visualise the observed distributions, heatmaps of counts from all 326 feet were generated for descriptive purposes. Statistical significance was defined as *p* < 0.05.

### Interpretation: Class Activation Mapping (CAM)

To interpret model predictions, we applied Gradient-weighted Class Activation Mapping (Grad-CAM), Score-based Class Activation Mapping (Score-CAM), and Layer-wise Class Activation Mapping (Layer-CAM) to visualise class-discriminative regions on plantar scan images. These methods generate activation maps that highlight spatial areas most influential to the model’s decisions, enabling pixel-level insight into model attention. Grad-CAM uses gradients from the final convolutional layer, while Score-CAM and Layer-CAM leverage input perturbation and intermediate feature activations, respectively, for enhanced localisation. The resulting heatmaps were overlaid on original images to assess whether the model focused on clinically relevant regions, such as the medial arch or heel. Visualising both correct and incorrect predictions allowed for qualitative evaluation of model plausibility and interpretability.

## Results

### Cohort characteristics

A total of 167 participants (334 feet) underwent 3D foot scanning and were included in the final dataset, based on the study’s inclusion criteria. After excluding 8 scans due to incomplete records or image quality issues, 326 plantar scans with corresponding labels were retained for analysis. All individuals underwent a clinical lower limb alignment assessment and 3D foot scanning. Demographic characteristics and label distributions are presented in Table 2.

**Table 2:** Distributions of anatomical categories and demographic factors.

Variables		Distribution			
Variable	Category	Counts per scan (feet)	Percent (%)	Male n (%)	Female n (%)
**Sex**	Male	190	58.3	—	—
	Female	136	41.7	—	—
**Arch height**	Neutral	113	34.7	59 (52.2)	54 (47.8)
	High arch	7	2.1	2 (28.6)	5 (71.4)
	Pes planus	206	63.2	129 (62.6)	77 (37.4)
**Hindfoot alignment**	Neutral	36	11.0	17 (47.2)	19 (52.8)
	Varus	27	8.3	9 (33.3)	18 (66.7)
	Valgus	263	80.7	164 (62.4)	99 (37.6)
**Knee alignment**	Neutral	53	16.3	28 (52.8)	25 (47.2)
	Varus	230	70.6	134 (58.3)	96 (41.7)
	Valgus	43	13.2	28 (65.1)	15 (34.9)

Percentages are calculated relative to the total number of scanned feet (N = 326). Male n (%) and Female n (%) represent the count and proportion of each sex within each alignment category.

### Statistical relationships between alignment categories

Because the dataset included bilateral scans from most participants (*N* = 326 feet from 167 enrolled participants), inferential analyses were conducted using one randomly selected foot per participant to preserve statistical independence. Filename-derived identifiers allowed 165 participants to be linked to their scan(s); therefore, the chi-square analyses included *N* = 165 participants.

The relationships between alignment categories are illustrated in Fig 3, using all 326 feet for descriptive purposes, whereas inferential tests used one foot per participant (*N* = 165). Arch type was not significantly associated with hindfoot alignment, $\chi^{2}(4,N=165)=5.03$, *p* = 0.285, Cramér’s *V* = 0.12, or with knee alignment, $\chi^{2}(4,N=165)=7.48$, *p* = 0.113, *V* = 0.15. The association between hindfoot and knee alignment was also not statistically significant, $\chi^{2}(4,N=165)=1.69$, *p* = 0.793, *V* = 0.07. Because several expected counts involving the high-arch category were < 5, Monte Carlo permutation tests for independence were performed as sensitivity analyses using 100,000 permutations. The permutation-based *p*-values (arch–hindfoot: *p* = 0.271; arch–knee: *p* = 0.105; hindfoot–knee: *p* = 0.804) were consistent with the asymptotic $\chi^{2}$ results. Overall, no statistically significant associations were identified among arch type, hindfoot alignment, and knee alignment in this sample.

**Fig 3:**
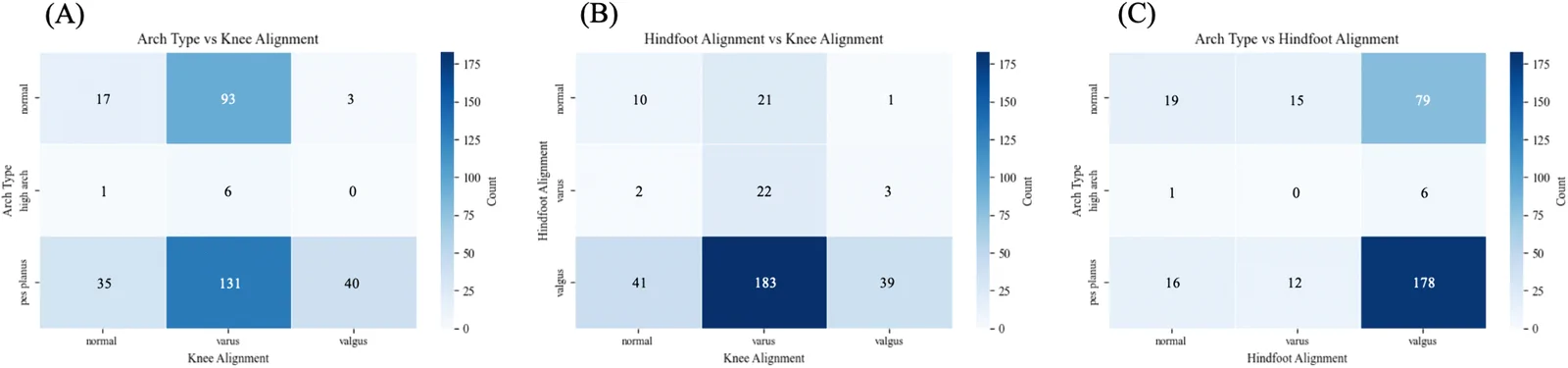
Heatmaps of observed counts for (A) Arch Type vs. Knee Alignment, (B) Hindfoot vs. Knee Alignment, and (C) Arch Type vs. Hindfoot Alignment; colours reflect count magnitude (darker = more). Cell labels show observed counts for all 326 feet; inferential analyses were performed using one foot per participant (*N* = 165).

### Model performance

We evaluated and compared the performance of four model configurations using accuracy, precision, recall, AUC, and F1 score on the held-out test set. Overall, our proposed method shows competitiveness across settings, and Fig 4 presents a radar chart comparison of different backbone architectures. It should be noted that severe class imbalance may reduce the reliability of precision, recall, and AUC metrics for these minority classes. Detailed confidence intervals (CIs) corresponding to all reported performance metrics are provided in S2 Table. Class-wise performance metrics for each task and model setting are provided in S3 Table.

**Fig 4:**
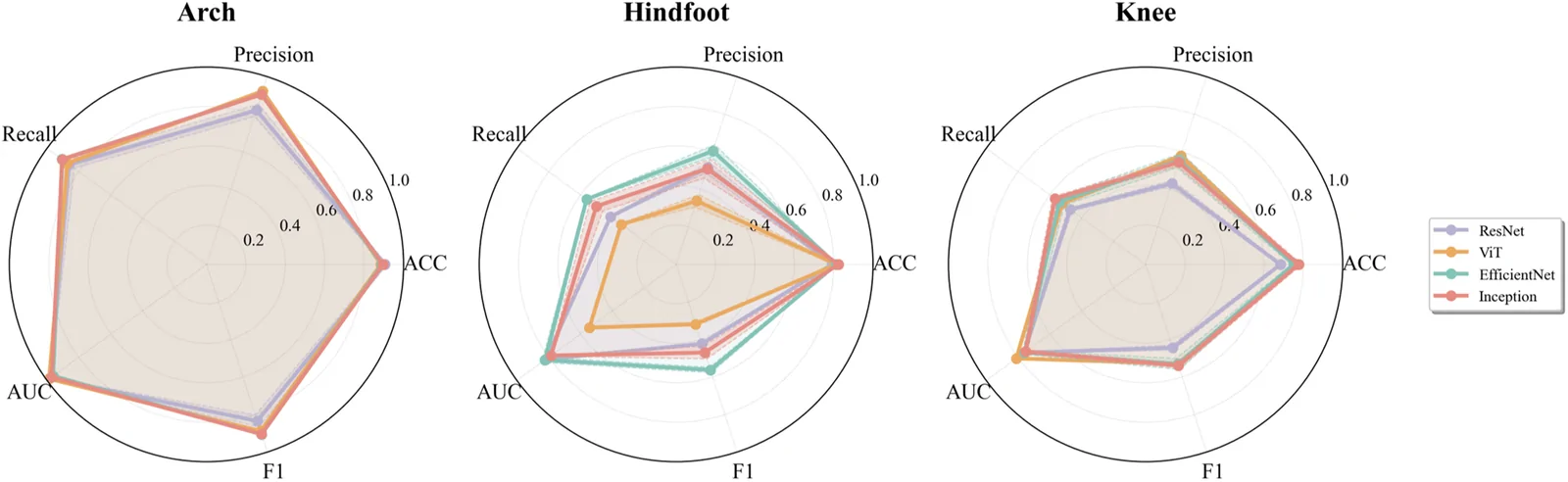
Radar chart comparing the performance of different backbone architectures across evaluation metrics for the three tasks using the proposed method. Inception (red) achieves higher performance across most metrics. Each axis represents a classification metric (precision, recall, accuracy, F1-score, AUC) on a scale of 0 to 1 (equivalent to 0–100%), with concentric gridlines at intervals of 0.2.

#### Arch type classification.

As shown in Table 3, arch type classification achieved the highest accuracy across three tasks. The high-arch class was sparsely represented, with seven cases in the full dataset and two in the held-out test set; therefore, class-specific performance estimates for this minority class should be interpreted as exploratory. The ViT image-only model outperformed all others, with accuracy, precision, recall, F1 score, and AUC all exceeding 0.94. Inception V3 with attention-based fusion also performed well, achieving 89% accuracy, an F1 score of 0.90, and an AUC of 0.98. In contrast, the non-fusion multimodal model achieved around 76% accuracy, and the numerical-only model was the least effective, with accuracy around 74%. These results suggest that visual features alone are highly discriminative for arch classification.

**Table 3:** Arch type classification performance.

Models		Performance metrics				
Setting	Encoder	Accuracy	Precision	Recall	AUC	F1
**Proposed**	EfficientNet	0.89±0.02	0.91±0.01	0.91±0.01	0.96±0.001	0.91±0.004
	**Inception**	**0.89 ± 0.04**	**0.90 ± 0.04**	**0.90 ± 0.04**	**0.98 ± 0.01**	**0.90 ± 0.04**
	ResNet	0.91±0.04	0.82±0.14	0.86±0.14	0.98±0.01	0.83±0.14
	ViT	0.89±0.02	0.92±0.03	0.87±0.04	0.99±0.01	0.89±0.03
**Without attention**	EfficientNet	0.81±0.04	0.59±0.13	0.60±0.14	0.21±0.05	0.59±0.13
	Inception	0.71±0.04	0.66±0.03	0.67±0.03	0.49±0.04	0.66±0.03
	ResNet	0.80±0.02	0.62±0.13	0.64±0.13	0.42±0.07	0.63±0.13
	ViT	0.76±0.03	0.52±0.12	0.52±0.11	0.71±0.09	0.52±0.11
**Image only**	EfficientNet	0.86±0.03	0.82±0.14	0.82±0.15	0.95±0.01	0.82±0.14
	Inception	0.89±0.04	0.90±0.04	0.90±0.04	0.98±0.01	0.90±0.04
	ResNet	0.89±0.01	0.88±0.06	0.88±0.04	0.97±0.01	0.87±0.05
	**ViT**	**0.94 ± 0.03**	**0.95 ± 0.03**	**0.95 ± 0.03**	**0.99 ± 0.01**	**0.95 ± 0.03**
**Morphology only**	Random Forest	0.77±0.04	0.56±0.13	0.54±0.07	0.86±0.01	0.55±0.09
	XGBoost	0.75±0.02	0.55±0.13	0.52±0.06	0.81±0.02	0.53±0.08
	MLP	0.73±0.05	0.54±0.15	0.51±0.09	0.83±0.04	0.52±0.11
	Logistic Regression	0.74±0.03	0.55±0.07	0.58±0.10	0.87±0.03	0.56±0.08

Values are reported as mean ± standard deviation. All metrics are macro-averaged across the three alignment classes.

#### Hindfoot alignment classification.

For hindfoot alignment prediction results illustrated in Table 4, the Inception V3 fusion model achieved an accuracy of 81% and showed competitive performance across the macro-averaged metrics. However, as shown by the class distribution in Table 2, valgus accounted for 80.7% of the dataset, meaning that a majority-class classifier would achieve the same accuracy. Hindfoot accuracy should therefore be interpreted cautiously and considered together with macro-averaged and class-wise performance metrics. A non-fusion multimodal model using EfficientNet achieved a slightly higher accuracy of 83%. In contrast, image-only and morphology-only models showed reduced performance, with accuracies near 75% and AUC values around 0.70. These results support the value of integrating morphological and visual cues, although the benefit varied by architecture and evaluation metric.

**Table 4:** Hindfoot alignment classification performance.

Models		Performance metrics				
Setting	Encoder	Accuracy	Precision	Recall	AUC	F1
**Proposed**	EfficientNet	0.81±0.01	0.60±0.14	0.56±0.02	0.82±0.08	0.56±0.06
	**Inception**	**0.81 ± 0.05**	**0.56 ± 0.17**	**0.46 ± 0.10**	**0.61 ± 0.10**	**0.47 ± 0.11**
	ResNet	0.79±0.03	0.52±0.12	0.41±0.02	0.82±0.01	0.42±0.03
	ViT	0.82±0.03	0.34±0.14	0.34±0.02	0.55±0.04	0.32±0.04
**Without attention**	**EfficientNet**	**0.83 ± 0.04**	**0.51 ± 0.05**	**0.47 ± 0.06**	**0.77 ± 0.03**	**0.47 ± 0.05**
	Inception	0.72±0.01	0.58±0.07	0.50±0.03	0.65±0.02	0.49±0.03
	ResNet	0.78±0.03	0.49±0.07	0.42±0.03	0.72±0.07	0.43±0.04
	ViT	0.68±0.01	0.40±0.07	0.38±0.04	0.60±0.02	0.38±0.04
**Image only**	EfficientNet	0.76±0.05	0.53±0.02	0.52±0.03	0.68±0.06	0.52±0.02
	Inception	0.74±0.05	0.50±0.10	0.47±0.10	0.66±0.09	0.46±0.08
	ResNet	0.73±0.07	0.55±0.10	0.51±0.08	0.64±0.10	0.51±0.08
	ViT	0.69±0.07	0.47±0.04	0.47±0.03	0.68±0.07	0.46±0.03
**Morphology only**	Random Forest	0.78±0.03	0.51±0.06	0.47±0.02	0.73±0.05	0.48±0.03
	XGBoost	0.80±0.04	0.55±0.09	0.52±0.07	0.76±0.07	0.53±0.08
	MLP	0.76±0.01	0.43±0.05	0.44±0.05	0.70±0.07	0.43±0.04
	Logistic Regression	0.67±0.06	0.44±0.05	0.47±0.07	0.72±0.11	0.44±0.06

Values are reported as mean ± standard deviation. All metrics are macro-averaged across the three alignment classes.

#### Knee alignment classification.

Knee alignment classification was the most challenging task, as shown in Table 5. The ViT without-attention model achieved the highest accuracy and macro-F1, while the Inception attention-fusion model showed closely comparable performance. Numerical-only models performed the worst, with accuracy near 63%, and non-fusion multimodal models exhibited inconsistent results. Attention-based fusion showed competitive performance in this task, where discriminative information may be more subtle and distributed across modalities.

**Table 5:** Knee alignment classification performance.

Models		Performance metrics				
Setting	Encoder	Accuracy	Precision	Recall	AUC	F1
**Proposed**	EfficientNet	0.75±0.05	0.55±0.16	0.54±0.12	0.76±0.06	0.53±0.12
	**Inception**	**0.76 ± 0.03**	**0.62 ± 0.08**	**0.60 ± 0.08**	**0.78 ± 0.05**	**0.60 ± 0.07**
	ResNet	0.68±0.04	0.43±0.07	0.47±0.05	0.76±0.03	0.44±0.06
	ViT	0.76±0.02	0.58±0.05	0.52±0.02	0.81±0.01	0.53±0.03
**Without attention**	EfficientNet	0.64±0.03	0.40±0.03	0.38±0.01	0.68±0.03	0.39±0.01
	Inception	0.76±0.03	0.46±0.03	0.57±0.02	0.71±0.02	0.50±0.03
	ResNet	0.74±0.05	0.47±0.03	0.52±0.04	0.88±0.03	0.49±0.03
	**ViT**	**0.78 ± 0.02**	**0.64 ± 0.03**	**0.60 ±0.03**	**0.87 ± 0.03**	**0.61 ± 0.03**
**Image only**	EfficientNet	0.73±0.04	0.66±0.07	0.65±0.05	0.81±0.02	0.62±0.05
	Inception	0.71±0.05	0.57±0.10	0.56±0.09	0.76±0.05	0.55±0.09
	ResNet	0.72±0.04	0.60±0.05	0.60±0.06	0.81±0.04	0.59±0.05
	ViT	0.74±0.02	0.54±0.05	0.50±0.04	0.74±0.05	0.51±0.04
**Morphology only**	Random Forest	0.67±0.04	0.50±0.06	0.50±0.05	0.71±0.05	0.49±0.05
	XGBoost	0.67±0.05	0.46±0.08	0.45±0.06	0.64±0.05	0.45±0.07
	MLP	0.64±0.06	0.48±0.05	0.46±0.04	0.67±0.03	0.46±0.04
	Logistic Regression	0.54±0.05	0.46±0.03	0.49±0.05	0.64±0.02	0.45±0.03

Values are reported as mean ± standard deviation. All metrics are macro-averaged across the three alignment classes.

Overall, attention-based fusion models—especially with Inception—showed competitive performance across tasks. The proposed method achieved higher descriptive performance than several morphology-only baselines for arch and knee alignment, while image-only models performed best for arch classification. For hindfoot and knee alignment, the benefit of multimodal fusion varied by architecture and metric. Participant-level overlap in the original split involved 42 participants between training and validation, 39 between training and test, and 9 between validation and test; 39 of 65 test scans were associated with participants also represented in training. Under strict participant-level splitting, morphology-only performance was slightly lower than under the original foot-level analysis, particularly for hindfoot and knee alignment, while moderate performance was retained for arch classification. Full results are provided in S4 Table.

### Model interpretability: Class activation mapping

Overall, Grad-CAM and Layer-CAM produced consistent results, with activation areas aligning well with expected anatomical features. Moreover, Score-CAM provided more localised and sharper attention maps, particularly in cases with clearer morphological variation, offering better anatomical precision in visualisation.

#### Grad-CAM, Score-CAM and Layer-CAM for arch.

Six representative cases (Case 1–6) were analysed for medial arch classification, as shown in Fig 5. The highlighted areas indicate the regions most influential in the model’s prediction.

**Fig 5:**
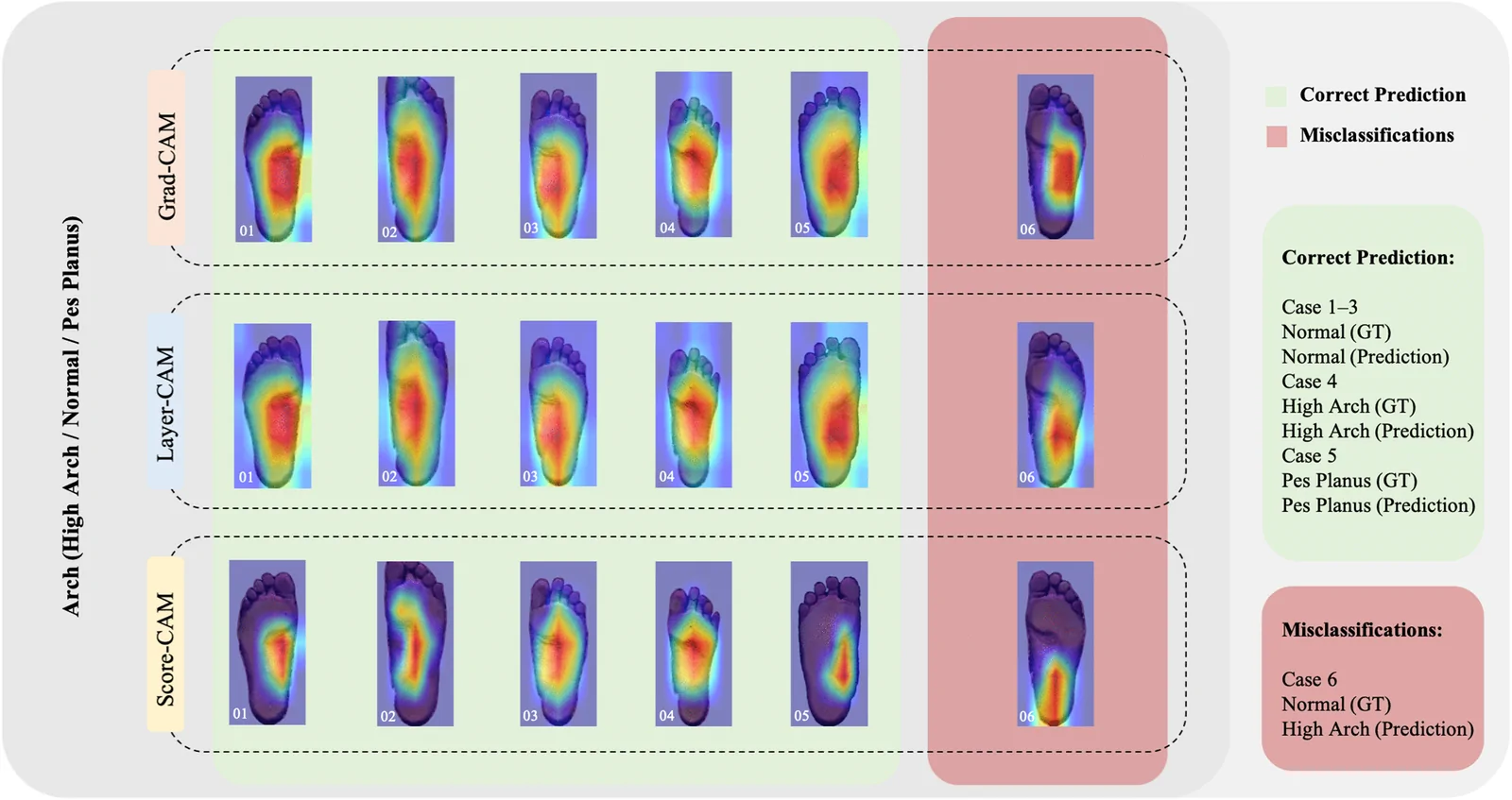
Visualisation of the arch task using Grad-CAM, Score-CAM, and Layer-CAM. The resulting heatmaps indicate the relative contribution of different plantar regions to the model’s prediction, with warmer colours denoting higher importance.

For correctly predicted cases, all three CAM methods consistently highlighted the medial longitudinal arch area, which is expected given its direct morphological visibility in plantar scan images. Notably, Case 4 (high arch) showed concentrated activation beneath the arch with clear medial compression, while Case 5 (pes planus) showed diffuse and broadened activation under the midfoot. In the misclassified Case 6, the activation map shifted away from the arch region and instead highlighted hindfoot areas, suggesting insufficient attention to the structurally relevant zone.

#### Grad-CAM, Score-CAM and Layer-CAM for hindfoot.

Six additional cases (Case 7–12) were examined for heel alignment, as shown in Fig 6.

**Fig 6:**
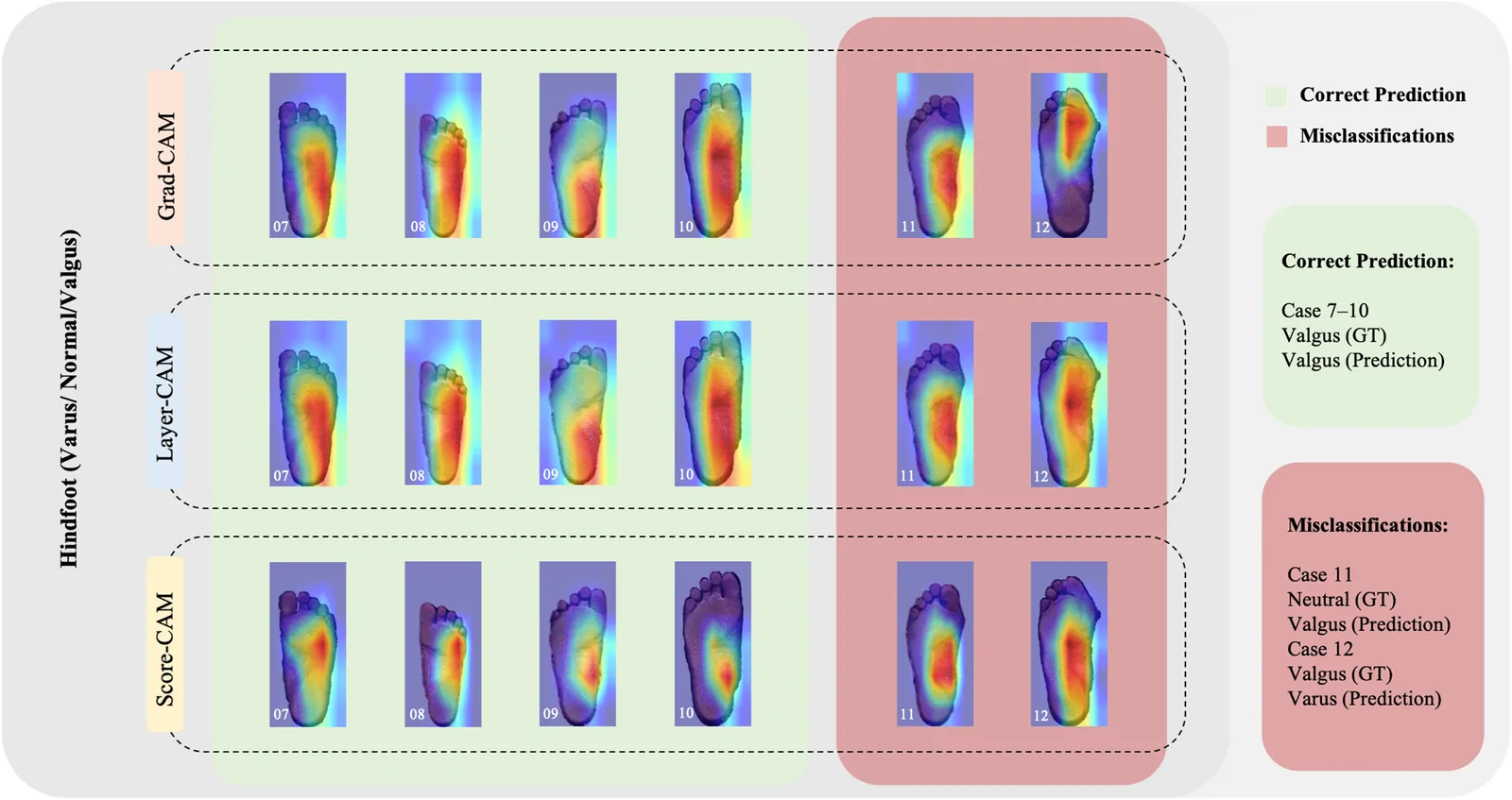
Visualisation of the hindfoot task using Grad-CAM, Score-CAM, and Layer-CAM. The resulting heatmaps indicate the relative contribution of different plantar regions to the model’s prediction, with warmer colours denoting higher importance.

For the correctly classified valgus heels, heatmaps from the three methods did not consistently concentrate on a single anatomical region. Cases 7 and 8 emphasised the lateral edge of the heel, while Cases 9 and 10 had heatmaps centred on the medial-posterior zone. In the two misclassified cases, activation maps diverged from heel anatomy and instead highlighted midfoot or arch regions.

#### Grad-CAM, Score-CAM and Layer-CAM for knee.

Cases 13–18 were assessed for predicted knee alignment, as shown in Fig 7.

**Fig 7:**
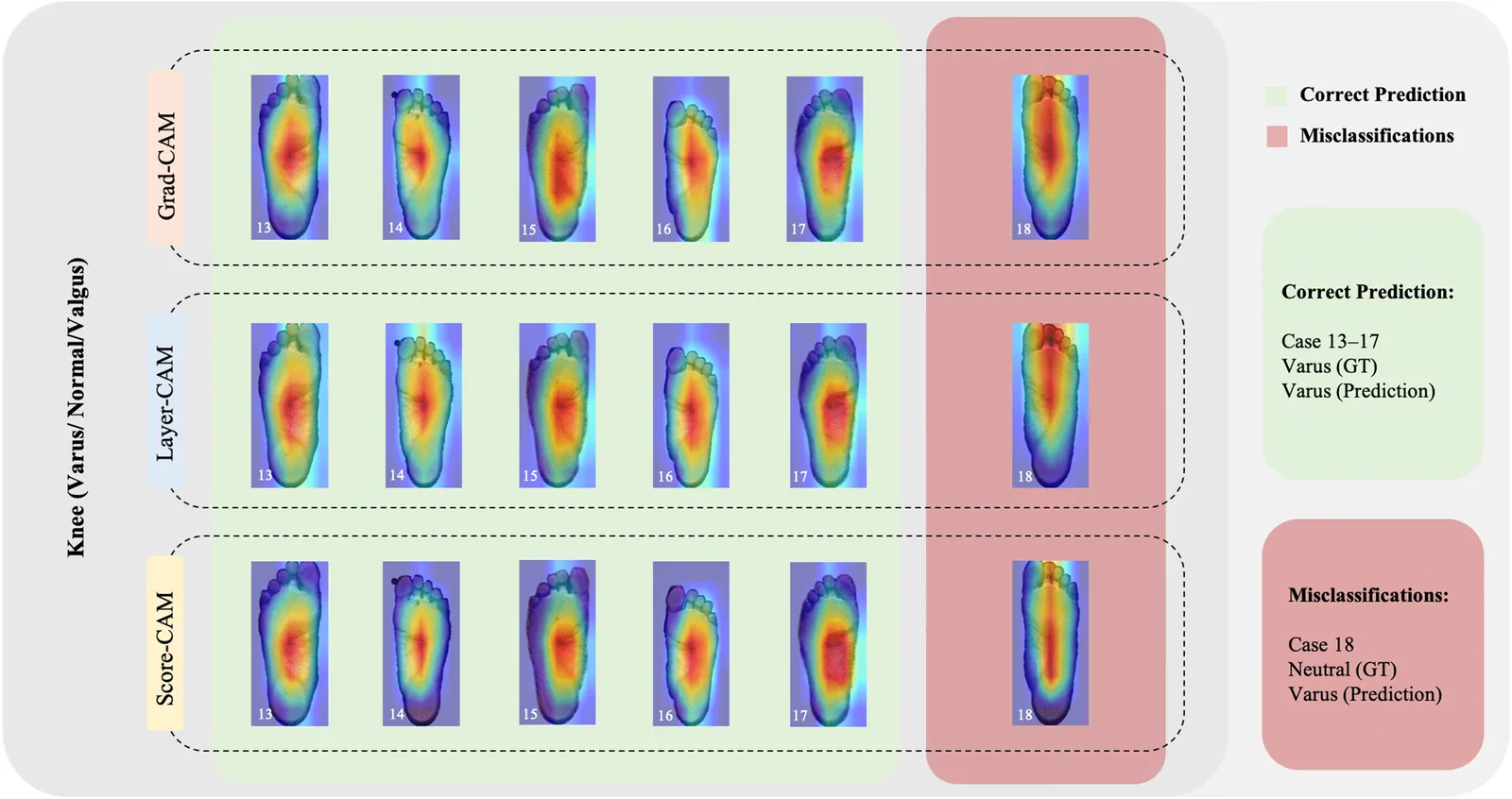
Visualisation of the knee task using Grad-CAM, Score-CAM, and Layer-CAM. The resulting heatmaps indicate the relative contribution of different plantar regions to the model’s prediction, with warmer colours denoting higher importance.

Unlike arch and heel tasks, knee alignment predictions were based on more subtle cues in the plantar image. In the correctly predicted varus cases, all three CAM methods consistently focused on the central plantar area, particularly the transverse arch and the region beneath the first metatarsophalangeal joint. In the misclassified Case 18, the activation was weak and diffusely spread, failing to highlight the typical forefoot turning point or lateral shift seen in varus patterns.

## Discussion

This study demonstrates how deep learning can be used to characterise the relationship between plantar foot morphology and clinically assessed lower-limb alignment (arch type, hindfoot alignment, and frontal-plane knee alignment) using weight-bearing plantar scan images and derived morphological features. The findings indicate potential utility for non-invasive screening and risk stratification in clinical and orthotic contexts, particularly for identifying individuals who may exhibit alignment patterns associated with elevated biomechanical risk.

From a biomechanical perspective, our findings can be interpreted within a kinetic-chain framework. Alterations at the hip or knee have been associated with measurable differences in foot structure and plantar loading [6,30], though the precise biomechanical mechanisms remain incompletely understood. Distal foot morphology may therefore reflect morphological correlates of proximal alignment, and learning-based models are well suited to capture complex, non-linear statistical associations between high-dimensional plantar data and alignment categories. Whether these associations reflect a true distal–proximal kinetic chain mechanism remains a hypothesis to be validated by future biomechanical studies. Prior work has demonstrated intersegmental coupling across the ankle, knee, hip, and pelvis in both open- and closed-chain conditions during gait [31]. Our results extend this literature by showing that static plantar images and derived morphology features contain useful signals for classifying lower-limb alignment patterns with moderate performance, noting that classification of hindfoot and knee alignment remains challenging, with F1 scores of 0.47 and 0.60, respectively, reflecting the difficulty of minority class prediction under class imbalance. Hindfoot alignment performance should be interpreted cautiously because valgus accounted for 80.7% of this real-world clinical dataset, which was not ideally balanced. The discrepancy between relatively high accuracy and lower macro-precision, macro-recall, and macro-F1 indicates persistent majority-class bias, particularly for the ViT-based model. This suggests that greater architectural complexity alone does not overcome limited minority-class representation, and larger, more balanced datasets are needed to improve class-balanced performance. More broadly, the benefit of attention-based fusion was task-dependent rather than uniform. For knee alignment, the ViT without-attention model slightly outperformed the proposed attention-fusion model, indicating that increased fusion complexity did not consistently improve performance. The lower accuracy for knee alignment (76%) than for hindfoot alignment (81%) may also reflect the greater anatomical distance between the plantar foot and the knee within the kinetic chain. Plantar morphology may carry a stronger and more direct signal for hindfoot alignment than for proximal knee alignment, where additional factors including soft tissue, muscle strength, and dynamic loading contribute to the observed coronal-plane position. Although radiographs remain the reference standard for precise coronal-plane measurements, they involve ionising radiation and do not fully reflect functional loading, supporting the potential value of non-invasive, image-based assessment for scalable screening. The broader concept of patient-specific “functional knee phenotypes” emphasises individualised patterns of alignment and load distribution [32]. Our framework is consistent with this view by modelling the foot–hindfoot–knee system as an integrated kinetic chain that can be assessed using distal plantar scanning. Future work integrating plantar imaging with unsupervised clustering could enable data-driven alignment phenotyping, supporting earlier identification and longitudinal monitoring of malalignment-related disorders.

In our study, CAM maps for knee alignment prediction often highlighted central plantar regions, including the transverse arch and the area beneath the first metatarsophalangeal joint, which is consistent with reports of altered forefoot loading in frontal-plane knee malalignment [33,34]. For hindfoot alignment, valgus deformity is commonly addressed by orthotists with support beneath the sustentaculum tali, and our model similarly localised this region. These observations are hypothesis-generating and suggest that plantar scan-based deep learning may warrant further investigation as a screening tool. However, as this is a cross-sectional observational study, any application to orthotic design or therapeutic intervention would require dedicated interventional studies before clinical translation can be considered.

The dataset size was modest and cross-sectional, which may limit generalisability. The cohort was recruited from a single centre in China and included participants aged 6–60 years. This broad age range reflects the real-world clinical population attending the centre but also introduces developmental heterogeneity and potential age-related confounding in plantar morphology and lower-limb alignment. The current dataset was not sufficiently powered for reliable age-stratified modelling; therefore, future studies should evaluate age-adjusted models and validate performance separately across paediatric, adolescent, and adult populations. Alignment labels were derived from standardised clinical physical examination rather than full-leg standing radiography; radiographic confirmation of a subset of cases is recommended for future work to strengthen ground truth validity [27,28]. This study used static weight-bearing plantar scans, which do not capture dynamic muscular compensations, ground reaction forces, or temporal loading patterns during locomotion. Future studies should incorporate dynamic plantar pressure measurements, together with more detailed demographic data, to better characterise functional loading patterns and improve assessment of potential confounding factors. Class activation maps provide spatial evidence of regions contributing to model predictions but do not establish causal biomechanical relationships; the highlighted regions therefore represent hypothesis-generating observations requiring dedicated validation studies. Class imbalance—particularly for high arch (2.1%) and hindfoot varus (8.3%)—may affect the reliability of minority-class metrics. For high arch, only seven cases were available in the full dataset, including two in the held-out test set; therefore, class-specific performance estimates for this category should be interpreted as exploratory. Although the foot-level split reflected the limb-specific screening unit, bilateral scans from the same participant are correlated and may have led to optimistic performance estimates. The participant-level sensitivity analysis showed reduced performance, particularly for proximal alignment tasks, and these findings should therefore be interpreted as preliminary. Future studies should expand sample size, include multisite recruitment and external validation, and incorporate longitudinal follow-up.

## Conclusion

This study presents an explainable multimodal deep learning framework for screening lower-limb alignment patterns – including arch type, hindfoot alignment, and frontal-plane knee alignment – using weight-bearing plantar scans and quantitative foot morphology features. Using a clinically annotated dataset, the proposed model achieved accuracies of 89%, 81%, and 76% for arch type, hindfoot alignment, and knee alignment and provided interpretable activation maps that highlighted anatomically meaningful plantar regions across tasks. By using non-invasive imaging and automated analysis, this approach offers a scalable digital method to support preliminary alignment assessment and musculoskeletal phenotyping, particularly in settings where radiographic evaluation is impractical or resource-limited. Our findings further indicate that plantar morphology carries informative signatures associated with proximal alignment, which may eventually support orthotic decision-making and earlier identification of alignment-related biomechanical risk factors. Future work should validate these findings in larger and more diverse cohorts, include external and multi-site testing, integrate dynamic modalities such as plantar pressure or gait measures, and assess longitudinal outcomes to understand alignment compensation and progression over time.

### Code availability

The code used for model development and model evaluation has been made publicly available in the associated GitHub repository: https://github.com/simonansm/FootAlignment.git. The repository includes scripts for model training and evaluation, label files, metadata, and a README file describing the project structure, software environment, data organisation, preprocessing workflow, model training procedure and evaluation pipeline.

## Supporting information

S1 Table Hyperparameter search space and optimal values for all deep learning models. All backbone models (EfficientNet, ResNet, ViT, Inception) were initialised with ImageNet pre-trained weights and fine-tuned end-to-end. Optimal values were selected via grid search across the search grid shown.
(XLSX)

S2 Table Model performance metrics with 95% CI across three tasks. Values are reported as mean (95% CI: lower, upper) for accuracy, precision, recall, AUC, and F1-score.
(XLSX)

S3 TablePer-class classification performance (mean ± SD) across three tasks, model settings, and labelled classes.Results are from 100 bootstrap iterations. Tasks: arch (Neutral / High arch / Pes planus), knee (Neutral / Varus / Valgus), and hindfoot (Neutral / Varus / Valgus). Results are shown for the proposed multimodal method, an image-only (Inception), and a morphology-only (Random Forest) baseline.(XLSX)

S4 TableMorphology-only classification performance under foot-level and participant-level splitting.Values are reported as mean ± standard deviation across repeated runs. Precision, recall, AUC, and F1-score are macro-averaged across the three classes.(XLSX)
